# Evaluation of the safety and immunogenicity of subcutaneous HX575 epoetin alfa in the treatment of anemia associated with chronic kidney disease in predialysis and dialysis patients 

**DOI:** 10.5414/CN109159

**Published:** 2017-08-02

**Authors:** Nicole Casadevall, Vladimir Dobronravov, Kai-Uwe Eckardt, Sehsuvar Ertürk, Liliya Martynyuk, Susanne Schmitt, Gregor Schaffar, Anita Rudy, Andriy Krendyukov, Marité Ode

**Affiliations:** 1 Service d’Immunologie et Hématologie Biologiques, Hopital Saint Antoine, Paris, France,; 2 Research Institute of Nephrology, 1st St Petersburg Pavlov State Medical University, St. Petersburg, Russia,; 3 Department of Nephrology and Hypertension, University of Erlangen-Nürnberg, Germany,; 4 Department of Nephrology, Ankara University School of Medicine, Ankara, Turkey,; 5 Subdepartment of Internal Medicine, Higher Educational Institution, I. Ya. Horbachevsky Ternopil State Medical University, Ternopil, Ukraine, and; 6 Hexal AG, Holzkirchen, Germany

**Keywords:** anemia, CKD, ESA, hemodialysis, dialysis

## Abstract

Aim: To assess the safety and immunogenicity of subcutaneous (SC) HX575 (epoetin-α) in dialysis- and nondialysis-dependent adult patients with chronic kidney disease (CKD). Methods: Open-label, single-arm, multicenter study in patients (n = 416) from Germany, Italy, Poland, Romania, Russia, Turkey, and Ukraine. Results: Mean (standard deviation (SD)) age was 52.3 (15.8) years, all patients were Caucasian, and similar proportions were male/female. 250 patients (60.1%) were erythropoiesis-stimulating agent (ESA)-naïve, and 166 (39.9%) were receiving ESA maintenance therapy at study start; mean (SD) on-study treatment duration with HX575 was 43.4 (15.8) weeks and 45.3 (13.7) weeks, respectively. Binding antierythropoietin (EPO) antibodies were detected by radioimmunoprecipitation (RIP) assay in 7 patients (1.7%; incidence 0.019); 5 of these were ESA-naïve at study entry. No patient developed neutralizing antibodies as determined in a cell-based epoetin neutralizing assay. Of the 7 patients with a positive binding anti-EPO RIP assay, 4 tested negative at later time points while continuing HX575 treatment. Three patients had low titers of anti-EPO antibodies at the last study assessment. There were no clinical signs of immunogenicity or hypersensitivity. Conclusions: SC HX575 was effective for correcting and maintaining correction of anemia, and the mean weekly dose remained stable over time.

## Introduction 

HX575 (Binocrit^®^, Sandoz (GmbH, Kundl, Austria) was approved in Europe in 2007 for chronic kidney disease (CKD)-related anemia by intravenous administration only. During the clinical development of HX575, the reference product (Erypo^®^/Eprex^®^, Janssen-Cilag/Ortho Biotech, Neuss, Germany) was contraindicated for subcutaneous (SC) use in CKD due to an increased risk of pure red cell aplasia (PRCA) [1]. Therefore, comparison was not possible, and SC route of administration to patients with CKD was not indicated in the initial approval of HX575. 

SC use of the reference product was subsequently reinstated, allowing a comparative study of HX575 and Erypo^®^/Eprex^®^ to be conducted [2]. The study was terminated prematurely due to two cases of neutralizing antibodies among patients treated with HX575. Apart from these two events, reported adverse events (AEs) were as expected for patients with CKD, and were similar in both treatment groups. 

A small number of individual syringes of two drug product batches were found to contain unusually high levels of protein aggregates at the end of the aborted clinical trial [3]. A thorough root-cause analysis identified aggregate formation due to increased tungsten levels as the possible explanation [3]; the source of these elevated tungsten levels was heat-resistant tungsten pins used to manufacture the glass syringes. Tungsten from this source has previously been implicated in the aggregation of other biopharmaceuticals [4, 5, 6]. The syringe type used to manufacture HX575 prefilled syringe was subsequently changed to a special low-tungsten form, and the European authorities approved a new study of SC HX575 in CKD-related anemia. Here we report the findings from this study. 

## Materials and methods 

This open-label, single-arm, multicenter study involved patients screened at 74 centers across 7 countries (Germany, Italy, Poland, Romania, Russia, Turkey, and Ukraine). The study assessed the immunogenicity of SC HX575 in the treatment of CKD-related anemia; the primary endpoint was incidence of antierythropoietin (EPO) antibody formation. Secondary objectives were to assess the safety of SC HX575 and to show that administration at least once per week adequately corrects or maintains the correction of anemia associated with CKD. 

### Patients 

Eligible patients were aged ≥ 18 years and had CKD-induced anemia (with or without dialysis) requiring erythropoiesis-stimulating agent (ESA) treatment. Anemia was defined as mean hemoglobin (Hb) concentration ≤ 11.0 g/dL for patients considered, for the purposes of the study, to be naïve to ESA therapy (did not receive ESA treatment during the 2 months prior to enrollment), or between 9.0 and 12.0 g/dL for patients receiving ESA therapy on at least 2 visits during the 4-week screening period. Inclusion criteria also included adequate iron substitution status (serum ferritin ≥ 200 μg/L and ≤ 800 μg/L for patients on dialysis and ≥ 100 μg/L and ≤ 800 μg/L for patients not on dialysis, and transferrin saturation ≥ 20% and ≤ 50%). 

Exclusion criteria included history of PRCA or anti-EPO antibodies; positive result for binding anti-EPO antibodies in a radioimmunoprecipitation (RIP) assay; lack of efficacy or loss of effect with a previous ESA therapy; history of use of any non-EU approved ESA; active gastrointestinal bleeding during the screening period; and any red blood cell or whole blood transfusion within the 3 months before visit 1. 

It was planned to include 360 patients, so that at least 240 would complete treatment with SC HX575 for 52 weeks, and ~ 300 patient-years of treatment with SC HX575 would be collected. Based on the Poisson distribution model for rare events, this would allow the detection of anti-EPO antibody formation or AEs with an incidence rate of 0.01 cases per patient year with a probability of 95%. 

### Study design 

Patient eligibility was assessed during a 4-week screening period, after which patients entered a 52-week treatment period. A 6-month safety follow-up was conducted for patients with binding, non-neutralizing anti-EPO antibodies. Patients were treated with SC HX575 at least once weekly. ESA-naïve patients began treatment on a starting dose of 25 IU/kg body weight 3 times weekly or 75 IU/kg body weight once per week from week 1 to week 5. Dose adjustments were possible after week 5. Patients on maintenance treatment with short-acting ESA were converted to treatment with HX575 at a weekly dose equivalent to that received before entering the treatment period. Patients on maintenance treatment with long-acting ESA (darbepoetin-α) were converted to HX575 by multiplying the weekly dose by 200. At baseline, the dosing frequency was changed to at least once a week. During treatment, the dose was individually titrated to maintain Hb concentrations in the range 10.0 – 12.0 g/dL. 

The evaluation of the immune response was based on a validated, highly-sensitive anti-EPO antibody-binding RIP assay and a validated cell-based neutralizing antibody assay performed at the sponsor’s laboratory. The first samples for antibody determination were collected at the first screening visit (visit –2) and analyzed before the patient entered the treatment period to ensure that patients positive for binding anti-EPO antibodies in the RIP assay were excluded before they received the first dose of study drug. The second sample was collected at visit 1, before the first dose of study drug was administered. Further samples were taken at visits 3, 5, 6, 7, 8, 9, 10, 11, 12, 13, 14, 15, at the end-of-study visit (visit 16), and at the safety follow-up visits. For patients on dialysis, blood was drawn before the dialysis session. All samples to be tested for anti-EPO antibodies were first analyzed in a screening RIP assay. When a result was above the screening cut-point, a confirmatory (specificity) RIP assay was performed. If the sample was positive for binding anti-EPO antibodies in the confirmatory RIP assay, it was additionally analyzed in a TF-1 cell (human erythroleukemic cell line) bioassay for determination of neutralizing anti-EPO antibodies. 

The applied anti-EPO antibody-binding RIP assay was highly specific with a sensitivity of 1.64 ng/mL. The assay detected low- as well as high-affinity immunoglobulin G and immunoglobulin M (neutralizing and non-neutralizing) anti-EPO antibodies [7]. In the screening RIP assay, a false-positive incidence rate of 5% was applied. To prove the specificity of the anti-EPO antibodies detected in the screening assay, screening-positive samples were reanalyzed in the confirmatory assay applying a final 1% false-positive rate. For semiquantification of the anti-EPO antibody response, the titer of confirmed binding anti-EPO antibody-positive samples was determined. 

Data were generally summarized using frequency tables (for categorical variables) and descriptive statistics comprising sample size, number of missing values, mean, standard deviation (SD), minimum, first quartile, median, third quartile, and maximum (for continuous variables). For all analyses by visit, an analysis visit was defined as a symmetrical window around the planned visit. For efficacy analyses, the mean (numerical variables) or the worst case (categorical variables) of all values within the analysis visit window was used. For safety analyses, the values closest to the planned visit day were used. 

Relevant ethics committees/institutional review boards approved the protocol, and the trial was conducted in accordance with the Declaration of Helsinki, International Council for Harmonization (ICH) Good Clinical Practice, and any applicable local regulations. All patients provided written, informed consent. The study was overseen by a steering committee that provided medical advice on presented questions or cases, and made appropriate recommendations based on the data. 

## Results 

**Figure 1.**
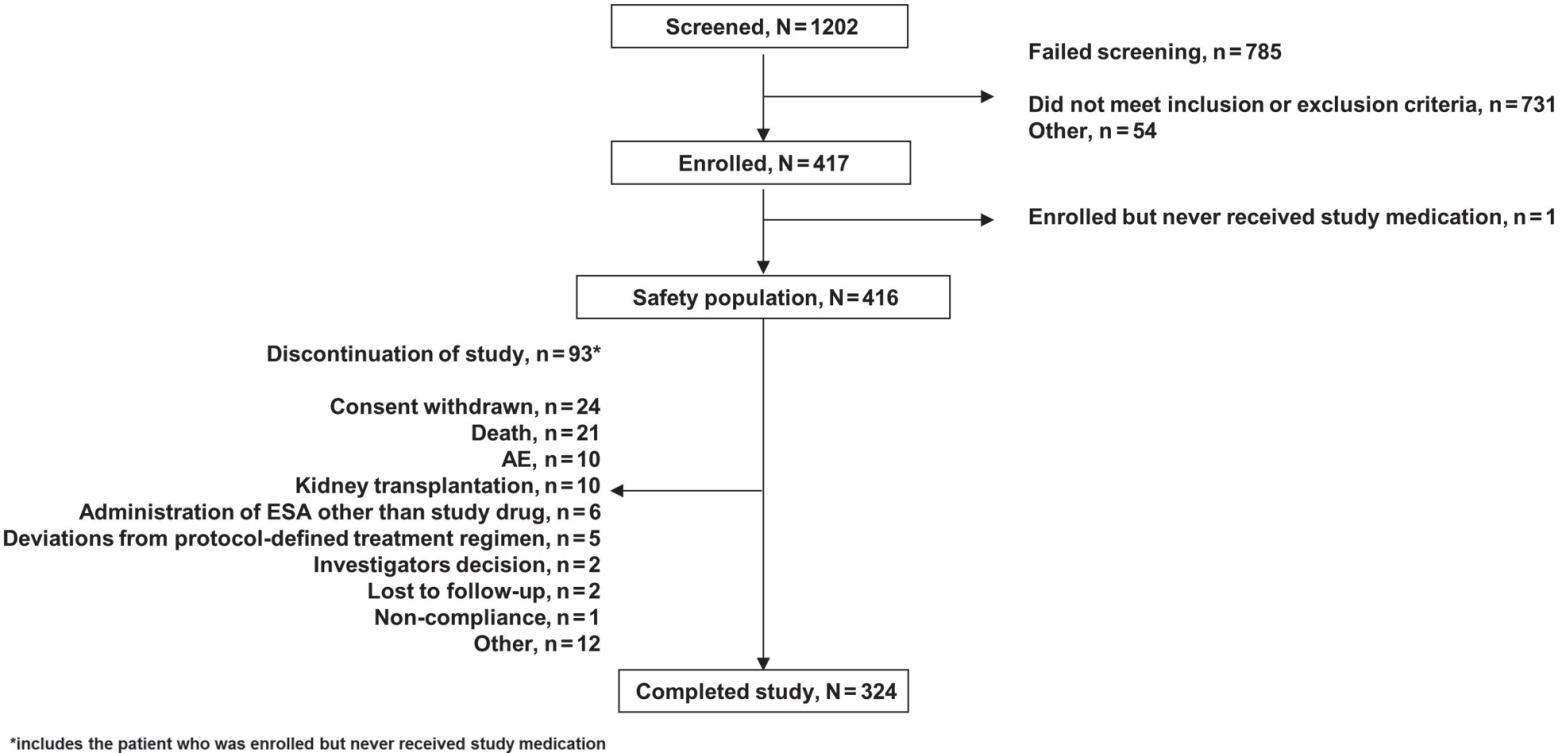
Patient disposition. AE = adverse event; ESA = erythropoiesis-stimulating agent.

Of the 1,202 patients screened, 417 were enrolled, and 416 received at least 1 dose of HX575 (Figure 1). Mean (SD) age was 52.3 (15.8) years, all patients were Caucasian, and there was a similar number of male (47.6%) and female (52.4%) patients (Table 1). A total of 250 patients (60.1%) were ESA-naïve, and 166 (39.9%) were already receiving ESA maintenance therapy with mostly non-study ESAs. Mean (SD) treatment duration of HX575 therapy was 43.4 (15.8) weeks and 45.3 (13.7) weeks in the ESA-naïve and maintenance groups, respectively. 

### Immunogenicity 

Of the 993 subjects tested in the RIP assay at first screening visit (before the first dose of study medication), 13 (1.3%; 95% confidence interval (CI): 0.7%, 2.2%) failed the screening due to a positive result; 11 of these patients were ESA-naïve, and 2 had been treated previously (before the screening assessment) with an ESA. 

During the study, binding anti-EPO antibodies were detected by the RIP assay in 7 patients (1.7%; incidence rate 0.019) (Table 2); 5 of these were ESA-naïve at study entry. Baseline characteristics of these patients were heterogeneous, with no obvious clinically-relevant patterns. For example, ages of the patients were 65, 53, 43, 22, 45, 66, and 71 years; 3 were male, and 4 were female. The primary cause of CKD was chronic glomerulonephritis (n = 3), diabetic nephropathy (n = 1), kidney abnormalities (n = 1), amyloidosis (n = 1), and hypertension (n = 1); 4 had stage 5 CKD and 3 had stage 4 CKD at study entry. The times between first exposure to study drug and the positive test result for these 7 patients were 224 days, 58 days, 86 days, 57 days, 115 days, 53 days, and 203 days, respectively. Of the 7 patients with a positive binding anti-EPO RIP assay, 4 tested negative in the assay at later time points during the study while still continuing HX575 treatment. Three patients had anti-EPO antibodies at their last study assessment, 1 patient at the end-of-study visit, and 2 patients during the safety follow-up (at 1 month and 6 months after the end of HX575 treatment). No patient developed neutralizing antibodies as tested in a cell-based assay. The detected binding antibodies had no clinically-evident impact on the treatment efficacy. There were no clinical signs of immunogenicity or hypersensitivity. 

There was no increase over the course of the 12-month study in the incidence of new cases of anti-EPO antibodies. The highest incidence was observed at visit 5 (week 8 – 10), when 3 patients (0.8%) were tested positive for the first time during the study. One further patient (0.3%) was tested positive for the first time at each of visits 6 (week 11 – 14), 7 (week 15 – 18), 10 (week 27 – 30), and 11 (week 31 – 34), respectively. In 2 of the anti-EPO antibody-positive patients, antibody levels increased, peaked, and then decreased again, but all on a low level. HX575 treatment was continued in all RIP-positive patients, and the titers of anti-EPO antibodies remained low in all 7 patients. 

### Hb levels 

**Figure 2.**
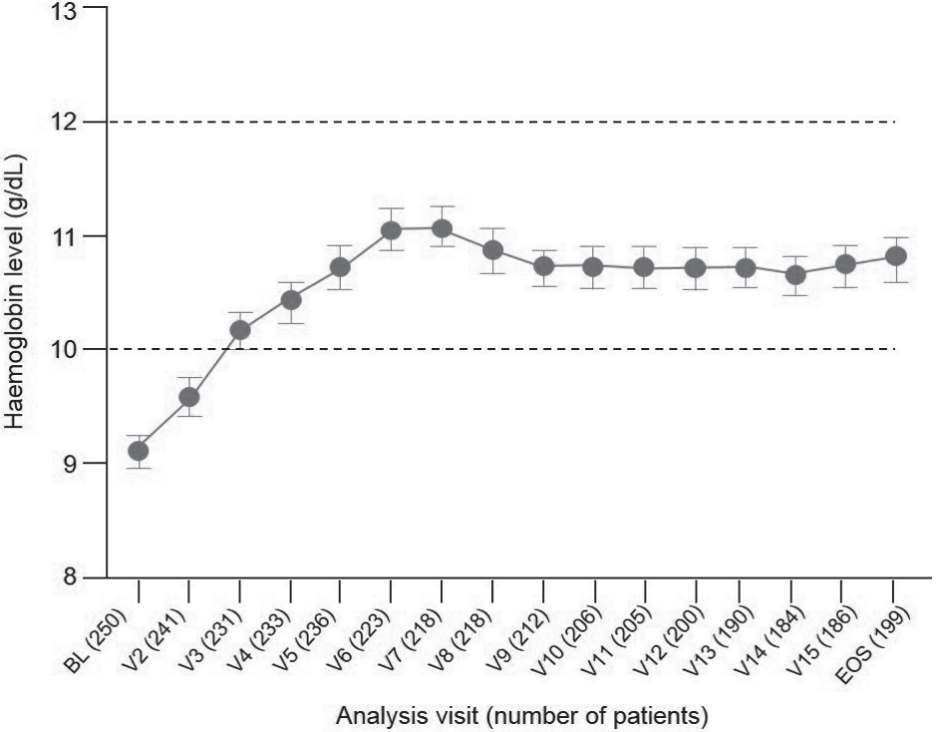
Mean (95% CI) hemoglobin concentrations during treatment in ESA-naïve patients (safety population, n = 250 of 416). BL = baseline; CI = confidence interval; ESA = erythropoiesis-stimulating agent; EOS = end of study; V = visit.

**Figure 3.**
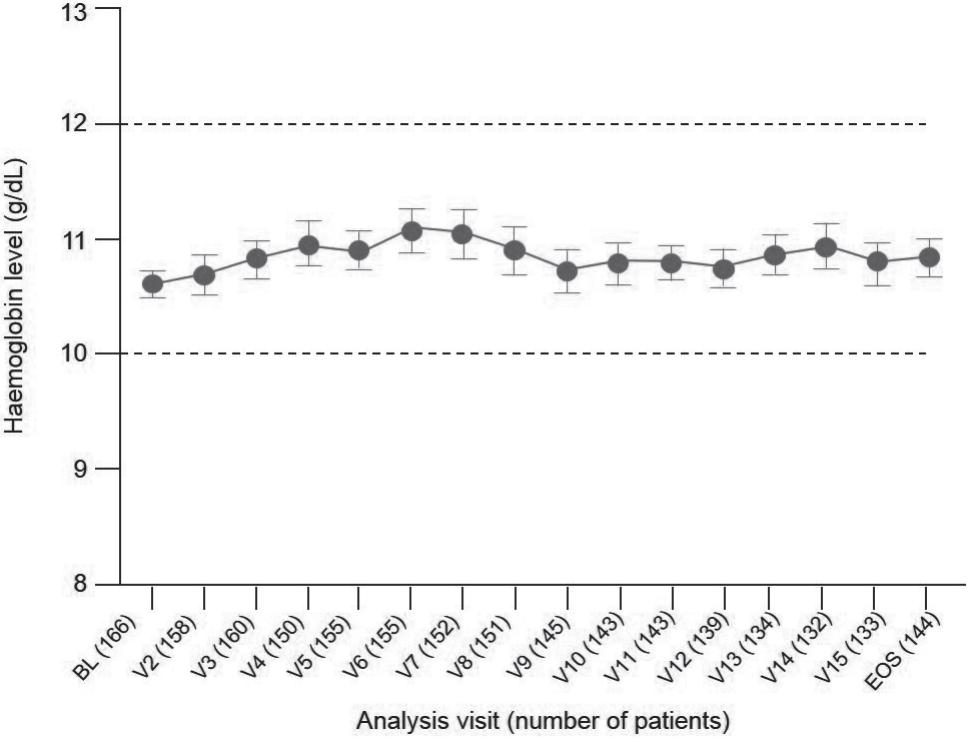
Mean (95% CI) hemoglobin concentrations during treatment in ESA-maintenance patients (safety population, n = 166 of 416). BL = baseline; CI = confidence interval; ESA = erythropoiesis-stimulating agent; EOS = end of study; V = visit.

Hb values over time are shown in Figure 2 for ESA-naïve patients and Figure 3 for ESA-maintenance patients. In ESA-naïve patients, the mean (SD) increase in Hb from baseline was 1.61 (1.60) g/dL at the end of the study. The percentage of patients within the Hb target range was 64.6% (95% CI: 57.6%, 71.1%) at the start of the maintenance phase (visit 10, week 27 – 30) and 57.8% (95% CI: 50.6%, 64.7%) at the end-of-study visit. In ESA-maintenance patients, the mean (SD) change in Hb from baseline was 0.22 (1.22) g/dL. The percentage of patients within the Hb target range was 76.5% (95% CI: 69.3%, 82.7%) at baseline and 70.1% (95% CI: 62.0%, 77.5%) at the end-of-study visit. 

### HX575 dose 

**Figure 4.**
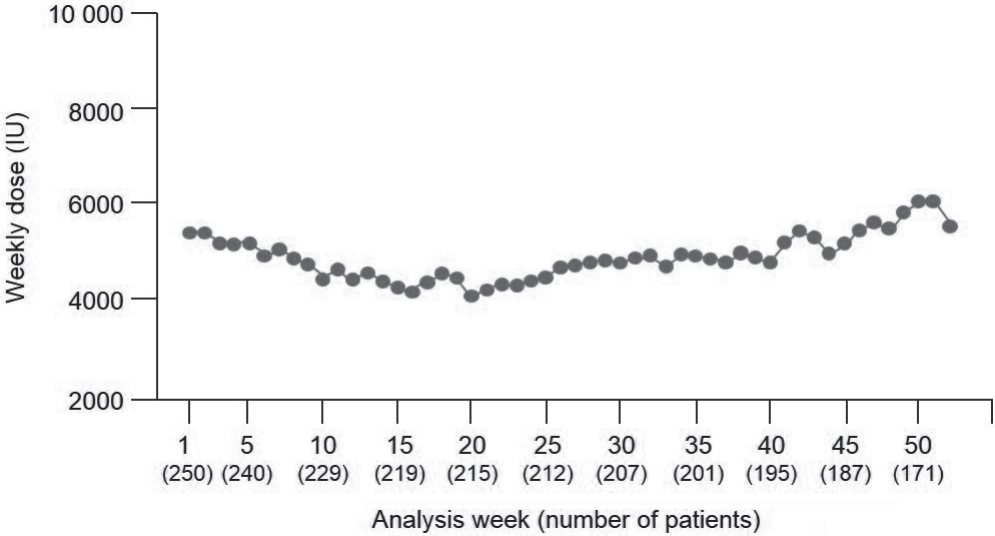
Mean weekly HX575 dose over time in ESA-naïve patients (safety population, n = 250 of 416). ESA = erythropoiesis-stimulating agent.

**Figure 5.**
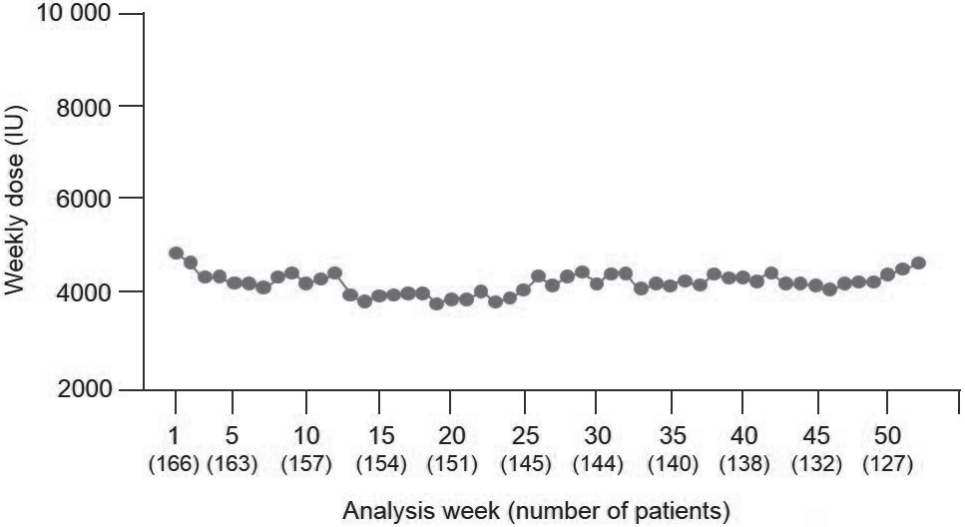
Mean weekly HX575 dose over time in ESA-maintenance patients (safety population, n = 166 of 416). ESA = erythropoiesis-stimulating agent.

For ESA-naïve patients, the mean weekly dose of HX575 was stable once they entered the maintenance phase (Figure 4). For ESA-maintenance patients, the mean weekly HX575 dose remained stable throughout the study period (Figure 5). In the safety population, 18.8% of all patients received iron therapy prior to the first administration of HX575. At week 1, iron-deficient patients (n = 61) were administered a mean (SD) dose of epoetin of 5,491.8 (1,822.3) IU, while iron-sufficient patients (n = 355) received 5,202.8 (2,253.3) IU. In both subgroups, the course over time followed a similar pattern to that observed for the overall population. 

### Safety and tolerability 

Reported AEs were as expected for the study population. An overview of treatment-emergent AEs (TEAEs) is shown in Table 3. 55.0% of patients reported TEAEs, and 5.3% of patients reported TEAEs considered by investigators to be related to the study drug. The most commonly reported TEAEs were chronic renal failure, hypertension, hyperkalemia, pneumonia, nasopharyngitis, and peripheral edema. Most TEAEs were mild or moderate in severity; 15.4% of patients reported severe TEAEs, mainly chronic renal failure (worsening). One severe TEAE (hyperkalemia) reported in 1 (0.2%) patient was considered related to HX575 treatment. 23.6% of patients reported serious TEAEs. The main TEAEs considered serious were chronic renal failure (worsening), peritonitis, pneumonia, and hypertension. A total of 21 patients died because of serious TEAEs, and 4 because of non treatment-emergent serious AEs. None of the deaths were considered related to the study drug. 

A total of 17 patients reported 18 TEAEs that led to study drug discontinuation. Two of these patients had TEAEs leading to study drug discontinuation with a suspected causal relationship to study drug. One patient reported an episode of nonserious chest pain that recovered after the study drug was withdrawn and the patient had received medication. The other patient reported a nonserious moderate event of hypertension that recovered once the study drug was stopped and the patient was treated with antihypertensive medication. 

46 patients reported 66 TEAEs related to thromboembolic events or malignancies. Three patients reported thromboembolic events with a suspected causal relationship to the study drug, as follows. One patient reported a nonserious event of peripheral swelling that recovered without changing HX575 treatment dose. Another patient reported a serious, moderate event of thrombosis of arteriovenous fistula that required hospitalization; the patient recovered with medication and without changing HX575 treatment dose. The third patient reported a nonserious, moderate episode of secondary hypertension that required medication and was ongoing at the end of the study; no change was made to HX575 treatment dose. Malignancies were reported in 2 patients: 1 was a fatal case of glioma and the other a case of adrenal mass for which the patient was hospitalized and then recovered. 

## Discussion 

SC administration of HX575 to patients (both ESA-naïve and maintenance) with CKD-related anemia did not lead to the development of neutralizing or clinically-relevant antibodies during the 12-month study period. HX575 administered at least once weekly was well tolerated and effective in this setting, independent of whether patients had previously been treated with ESA. 

The previous study assessing SC administration of HX575 was ended prematurely following occurrence of 2 cases of neutralizing antibodies [2]. A subsequent root-cause analysis identified HX575 aggregation due to increased tungsten levels in some prefilled syringes [3], and the type of syringes used to manufacture HX575 prefilled syringes was subsequently changed. The current study confirms that, following this change of syringe type, the immune potential of HX575 when administered SC is low. 

Of the 416 treated patients, 7 tested positive for binding anti-EPO antibodies. Four of these 7 patients were negative at later time points in the study. It is interesting to note that at the first screening visit, before study treatment was started, 13 patients (11 ESA-naïve and 2 ESA pretreated) of 993 tested (1.3%) were positive for anti-EPO antibodies; the origin of these antibodies is unclear, and further investigation is warranted. It should be noted that, given the definition of ESA-naïve used in the study (no ESA treatment during the 2 months prior to enrollment), some of these patients may not be ESA-naïve from an immunological perspective. In the 3 patients with binding anti-EPO antibodies at the last study assessment, all detected antibodies were of low titers and were non-neutralizing. There were no clinical signs of immunogenicity or hypersensitivity. 

Others have reported detectable levels of non-neutralizing anti-ESA antibodies in ESA-naïve patients [8, 9]. In one report, antibody data were compiled for over 6,000 patients from clinical studies and the postmarketing setting [8]. Pre-existing, non-neutralizing anti-ESA antibodies were found in 6% of subjects from clinical studies in nephrology, oncology, and congestive heart failure; in this same report, 2.3% of subjects developed binding, non-neutralizing antibodies after ESA treatment. It is important to recognize that the detection of antibodies to EPO is dependent to some degree on the immunoassay platform used [10]. 

Applying the definition of persistence suggested by Shankar et al. [11], the anti-EPO antibody response could be considered persistent in 5 patients and transient in 2 patients; this is because the antibodies were detected at 2 or more time points, with the first and last positive samples separated by more than 16 weeks, or with 1 positive sample less than 16 weeks before a negative last sample. However, given the very high sensitivity of the RIP assay with a 1% false-positive rate, and considering the low antibody titers of the positive samples with continued SC HX575 treatment, a persistent antidrug antibody response was excluded, and the anti-EPO antibody response is considered to be transient in all 7 RIP-positive patients. This is also supported by the comparable binding anti-EPO antibody positive rate determined in naïve and pretreated patients during the screening phase of the study. 

Several patient-related factors have been suggested that may play a role in the development of an immune response to epoetins. These include the presence of concomitant illness, a patient’s immune status, and a patient’s genetic background [12, 13, 14]. In addition, a high number of elderly males has been noted among cases of epoetin-related PRCA [15]. Taking age, gender, and other factors such as comorbidities and concomitant medications (data not shown) into consideration, no obvious link or pattern was noted among the RIP-positive patients in the present study. 

HX575 was effective, regardless of whether patients had previously received ESA therapy. ESA-naïve patients increased their Hb concentration, and in those on ESA maintenance therapy, the percentage of patients with Hb concentrations within the target range was similar from baseline to the end of the study. The mean HX575 dose was stable in maintenance patients throughout the study, and in ESA-naïve patients during the maintenance phase. Iron-deficient patients in the safety population received a higher mean dose of ESA than iron-replete patients, highlighting the need to compensate for the lower iron status. 

SC-administered HX575 in prefilled syringes was well tolerated; reported AEs were consistent with the previously-reported safety profile of the study drug [16] and the patient population. 

In summary, binding, non-neutralizing antibodies were detected with highly sensitive assays in 1.7% of patients with anemia receiving SC HX575, but there was no evidence of a clinically-relevant impact. HX575 administered SC at least once a week was effective in managing anemia in CKD patients, regardless of whether they had previously been treated with ESA. These results suggest that SC administration of HX575 is a suitable treatment option for CKD patients with anemia. 

## Acknowledgment 

The authors thank all investigators, patients, and their families who participated in the study. We also thank Antje Speer and Gabor Stiegler for their contribution to the study. Medical writing support was provided by Tony Reardon of Spirit Medical Communications Ltd, and supported by Sandoz International GmbH/Hexal AG. 

## Conflict of interest 

NC has received consulting fees from Sandoz, Novartis, and Shire; and lecture fees from Novartis. VD has received consulting fees from Hexal AG, and lecture fees from Alexion and Fresenius Kabi. KUE has received consulting fees from Akebia, Amgen, Johnson & Johnson, and Sandoz/Hexal; lecture fees from Chugai and Roche; and grant support from Amgen and AstraZeneca. SE and LM have no potential conflicts of interest to declare. SS, MO, GS, AR, and AK are employees of Sandoz/Hexal AG. 

**Table 1.** Baseline demographic and clinical characteristics (safety population, n = 416).

Parameter	
Mean age, years (SD)	52.3 (15.8)
Sex, n (%) Male Female	198 (47.6) 218 (52.4)
Race, n (%) Caucasian	416 (100.0)
Mean height, cm (SD)	166.9 (9.4)
Mean weight, kg (SD)	70.6 (15.9)
Mean BMI, kg/m^2^ (SD)	25.3 (5.07)
Mean Hb, g/dL (SD) Iron-replete (n = 355) Iron-deficient (n = 61)	9.81 (1.288) 9.25 (1.262)
Patients receiving iron therapy, n (%)	78 (18.8)
Dialysis status, n (%) Dialysis Predialysis	252 (60.6) 164 (39.4)

BMI = body mass index; Hb = hemoglobin; n = number of patients; SD = standard deviation.

**Table 2.** Incidence of positive test results for anti-EPO antibodies during the treatment period (safety population, n = 416).

Assay/result	n	% (95% CI)	IR treatment	IR risk
Binding ADA/positive	7	1.7 (0.7, 3.4)	0.019	0.019
Neutralizing ADA/positive	0	–	–	–

Percentages based on number of patients with any non-missing result. ADA = anti-drug antibody; CI = confidence interval; EPO = erythropoietin; IR = incidence rate (IR treatment is based on duration of treatment period, IR risk on duration from first day of treatment to first event or censored at last day of treatment); n = number of patients.

**Table 3.** AEs overview (safety population, n = 416).

	HX575		
	n (%)^a^	Events	IR treatment^b^
TEAEs	229 (55.0)	722	0.593
Related TEAEs	22 (5.3)	35	0.057
Treatment-emergent SAEs	98 (23.6)	172	0.254
Related treatment-emergent SAEs	4 (1.0)	4	0.010
TEAEs of special interest^c^	46 (11.1)	66	0.119
Related TEAEs of special interest^c^	3 (0.7)	3	0.008
TEAEs leading to study drug discontinuation	17 (4.1)	18	0.044
TEAEs leading to death	21 (5.0)	25	0.054

^a^Patients with at least 1 AE; ^b^relative to patient years under treatment; ^c^malignancies and thromboembolic events. AE = adverse event; IR = treatment, incidence rate relative to patient years under treatment; n = number of patients; SAE = serious adverse event; TEAE = treatment-emergent adverse event.
